# Trimethoprim resistance in surface and wastewater is mediated by contrasting variants of the *dfrB* gene

**DOI:** 10.1038/s41396-023-01460-7

**Published:** 2023-06-27

**Authors:** David Kneis, Claudèle Lemay-St-Denis, Stella Cellier-Goetghebeur, Alan X. Elena, Thomas U. Berendonk, Joelle N. Pelletier, Stefanie Heß

**Affiliations:** 1 TU Dresden, Institute of Hydrobiology, 01062 Dresden, Germany; 2 PROTEO, The Québec Network for Research on Protein, Function, Engineering and Applications, Quebec, QC Canada; 3 CGCC, Center in Green Chemistry and Catalysis, Montréal, QC Canada; 4 Department of Biochemistry & Molecular Medicine, University of Montréal, Montréal, QC H3T 1J4 Canada; 5 Chemistry Department, University of Montréal, Montréal, QC H2V 0B3 Canada; 6 TU Dresden, Institute of Microbiology, 01062 Dresden, Germany

**Keywords:** Water microbiology, Antibiotics, Metagenomics

## Abstract

Trimethoprim (TMP) is a low-cost, widely prescribed antibiotic. Its effectiveness is increasingly challenged by the spread of genes coding for TMP-resistant dihydrofolate reductases: *dfrA*, and the lesser-known, evolutionarily unrelated *dfrB*. Despite recent reports of novel variants conferring high level TMP resistance (*dfrB10* to *dfrB21*), the prevalence of *dfrB* is still unknown due to underreporting, heterogeneity of the analyzed genetic material in terms of isolation sources, and limited bioinformatic processing. In this study, we explored a coherent set of shotgun metagenomic sequences to quantitatively estimate the abundance of *dfrB* gene variants in aquatic environments. Specifically, we scanned sequences originating from influents and effluents of municipal sewage treatment plants as well as river-borne microbiomes. Our analyses reveal an increased prevalence of *dfrB1, dfrB2*, *dfrB3*, *dfrB4*, *dfrB5*, and *dfrB7* in wastewater microbiomes as compared to freshwater. These gene variants were frequently found in genomic neighborship with other resistance genes, transposable elements, and integrons, indicating their mobility. By contrast, the relative abundances of the more recently discovered variants *dfrB9*, *dfrB10*, and *dfrB13* were significantly higher in freshwater than in wastewater microbiomes. Moreover, their direct neighborship with other resistance genes or markers of mobile genetic elements was significantly less likely. Our findings suggest that natural freshwater communities form a major reservoir of the recently discovered *dfrB* gene variants. Their proliferation and mobilization in response to the exposure of freshwater communities to selective TMP concentrations may promote the prevalence of high-level TMP resistance and thus limit the future effectiveness of antimicrobial therapies.

## Introduction

Trimethoprim (TMP) is a bacteriostatic antibiotic of the diaminopyrimidine class. Of synthetic origin, as opposed to naturally derived antibiotics such as penicillins, it inhibits the growth of a range of aerobic bacteria including several Gram-positive and -negative pathogens [1]. First introduced in the 1960s [2], TMP is mainly employed in the treatment of urinary tract infections [3] as a single medication or in combination with sulfamethoxazole (co-trimoxazole) to treat a wide range of bacterial infections. In susceptible bacteria, TMP inhibits the enzymatic reduction of dihydrofolate to tetrahydrofolate by the bacterial FolA dihydrofolate reductase (DHFR) [1]. This in turn disrupts DNA synthesis through the suppression of purine and pyrimidine production [4], thus abrogating microbial proliferation. TMP and co-trimoxazole are included in the recent list of essential medicines maintained by the World Health Organization [5].

According to recent studies, TMP resistance is common in urinary tract infections caused by, for example, *Escherichia coli* or *Klebsiella* spp. [6]. The observed prevalence of resistance may reflect the selective pressure due to frequent TMP administration [7]. However, other factors can promote the prevalence of TMP resistance, such as co-selection [8] in cases where resistance genes targeted at other antibiotics are found on the same genetic element such as transposons [9] or multi-resistance plasmids [2]. An increase in the abundance of TMP resistance in clinically relevant pathogens has been identified by several studies [10, 11] and a reversal of resistance through the sole reduction of TMP administration is unlikely to be achievable [12].

At the mechanistic level, resistance may be due to mutations in the primary *folA* gene resulting in reduced TMP susceptibility [13], overproduction of such enzymes, selection toward a limited uptake or binding of TMP [14], or the action of efflux pumps [15]. The most important mechanism, however, appears to be the acquisition of type A dihydrofolate reductases (DfrA) which represent TMP-resistant variants of FolA [16, 17]. Unlike the primary FolA enzyme found in wild-type populations, DfrA variants allow bacterial cells to sustain DNA synthesis in the presence of moderate TMP levels [18]. Genes coding for DfrA were found on mobile genetic elements as early as the 1970s [9, 19, 20] and the clinical relevance of horizontal transmission of *dfrA* has been confirmed by case reports [21]. The *dfrA* genes emerged from both recent mutations in TMP-sensitive *folA* genes and pre-existing TMP-resistant *folA* genes, followed by mobilization in the resistome [17]. The CARD database [22] currently lists 57 members of the *dfr* gene family coding for TMP-resistant dihydrofolate reductases, many of which have been found on plasmids and within integron gene cassettes [23–25]. Most of these genes fall into the *dfrA* sub-family.

The family of type B dihydrofolate reductases (DfrB) provides an alternative mechanism for TMP resistance. The *dfrB* genes share no significant sequence nor structural similarities with FolA and DfrA, indicative of a distinct evolutionary origin for this family [26, 27]. Although *dfrB* were originally identified in clinical samples [28, 29], their prevalence is currently unknown, as they have not been routinely searched for whether by PCR methods in earlier decades or more recently by genomic identification [6]. Hence, the limited amount of available data on the distribution of *dfrB* genes has impeded our understanding of their emergence.

Only recently, two new variants of the *dfrB* family, *dfrB10* and *dfrB11* have been discovered and confirmed in their provision of high-level phenotypic TMP resistance [28]. Since then, ten additional variants labeled *dfrB12* to *dfrB21* have been identified in genome sequences of isolates and metagenomes based on homology, followed by experimental validation [30]. Some of these *dfrB* sequences originate from environmental samples where TMP exposure is supposed to be marginal. This gave rise to the hypothesis that the original selective advantage conferred by *dfrB* genes may be unrelated to TMP resistance [30]. However, this hypothesis was based on identification of *dfrB* sequences in few samples, which is why the possibility of contamination effects [31–33] cannot be excluded. Verification of the hypothesis was further hampered by data heterogeneity with regard to the origin of samples, the source of DNA (isolates vs. metagenomes), and sparse metainformation.

In this study, we provide the first large-scale, statistically verified analysis of the prevalence of this emerging source of high TMP resistance by evaluating shotgun metagenomic sequences of both freshwater and wastewater environments (324 samples, 7 ×10^9^ high-quality sequences). We provide quantitative estimates on the abundance of *dfrB* genes in aquatic environments with and without strong human impact along with indicators of gene mobility. By comparing the distribution of particular gene variants across contrasting environments, our work contributes to the identification of the origin of *dfrB* which is a key to understanding the epidemiology of novel TMP resistance. In particular, our study highlights the possible role of environmental bacteria in the emergence of resistance to synthetic antimicrobials where evolutionary adaptation to naturally produced analogues did not take place.

## Materials and methods

### Origin and characteristics of the analyzed metagenomes

For this study, we analyzed publicly available metagenomic DNA sequences of freshwater and wastewater bacterial communities downloaded from SRA (sequence read archive; https://www.ncbi.nlm.nih.gov/sra). The picked sequences exclusively represent DNA fragments generated by the shotgun technique which does not involve amplification of particular targets, that is, the “selection” attribute of the sequencing library had to be “random”. For consistent processing and comparability, only samples that were sequenced on an Illumina instrument in paired-end layout were included. The latter integrate wastewater and freshwater samples from the temperate zone such that, in each category, at least five different countries are represented. The analyzed data consist of 324 distinct samples from the US, UK, New Zealand, China, Canada, Sweden, and Germany (Table 1). Full accession and metainformation is provided in the supplement Table S1. In total, the data comprise about 7 × 10^9^ high quality read pairs. Freshwater samples were subdivided into two categories (unpolluted, polluted) based on the exposure of the respective sampling sites to effluents from municipal wastewater treatment plant effluents where sufficient on-site information was available (dataset “q” in Table 1). A further distinction was made between water and sediment samples.

**Table 1:** Characteristics of the analyzed data sets.

Matrix	Country	Study	Samples	Sites	Reads	Bases	Read length	16S
WWTP influent	CD	PRJNA768945 (a)	8	1	3e + 07	6e + 09	208	1e + 04
WWTP influent	CN	PRJNA824545 (p)	4	1	7e + 07	2e + 10	230	9e + 04
WWTP influent	DE	PRJNA524094 (d)	14	2	2e + 08	6e + 10	261	5e + 05
WWTP influent	DE	PRJNA942078 (r)	14	9	4e + 08	1e + 11	259	1e + 06
WWTP influent	NZ	PRJNA904380 (e)	9	1	1e + 08	4e + 10	278	5e + 05
WWTP influent	US	PRJNA683044 (i)	8	1	1e + 08	4e + 10	269	4e + 05
WWTP influent	US	PRJNA691978 (n)	3	1	2e + 07	5e + 09	211	7e + 04
WWTP effluent	CD	PRJNA768945 (a)	8	1	3e + 07	6e + 09	211	1e + 04
WWTP effluent	CN	PRJNA824545 (p)	4	1	8e + 07	2e + 10	241	4e + 04
WWTP effluent	DE	PRJNA524094 (d)	13	2	2e + 08	6e + 10	273	1e + 05
WWTP effluent	DE	PRJNA892917 (q)	9	1	2e + 08	5e + 10	263	1e + 05
WWTP effluent	NZ	PRJNA904380 (e)	9	1	1e + 08	4e + 10	281	2e + 05
WWTP effluent	SE	PRJEB14051 (g)	6	3	8e + 07	1e + 10	144	9e + 04
WWTP effluent	UK	PRJNA529503 (h)	8	1	1e + 08	3e + 10	241	2e + 05
WWTP effluent	US	PRJNA683044 (i)	10	1	2e + 08	5e + 10	270	1e + 05
River water	CN	PRJNA559231 (b)	30	27	7e + 08	2e + 11	271	8e + 05
River water	CN	PRJNA798157 (c)	3	3	2e + 08	4e + 10	257	2e + 05
River water	DE	PRJNA892917 (q)	14	9	4e + 08	9e + 10	252	2e + 05
River water	NZ	PRJNA668816 (f)	2	2	6e + 07	3e + 10	449	8e + 04
River sediment	CN	PRJNA559231 (b)	32	25	8e + 08	2e + 11	261	4e + 05
River sediment	CN	PRJNA798157 (c)	3	3	2e + 08	5e + 10	243	8e + 04
River sediment	DE	PRJNA892917 (q)	68	18	2e + 09	4e + 11	257	9e + 05
River sediment	NZ	PRJNA668816 (f)	6	2	1e + 08	6e + 10	426	8e + 04
River sediment	UK	PRJNA529503 (h)	7	1	9e + 07	2e + 10	250	5e + 04
River sediment	US	PRJEB23134 (m)	16	1	2e + 08	6e + 10	268	1e + 05
River sediment	US	PRJNA795480 (k)	16	15	1e + 08	2e + 10	232	6e + 04

Studies are denoted by their SRA identifiers; lower case letters were added for convenient references within this publication. The number of reads, bases, and the average read length represent the state after quality-trimming and merging of read pairs. The number of 16 S rRNA gene copies is indicated in column “16 S”. See Table S1 in the supplementary material for full metainformation on individual samples, including SRA run accession numbers.

### Bioinformatics

#### Processing of raw sequence data and short read analyses

All samples were processed through the same bioinformatics pipeline involving the simultaneous removal of purely technical sequences and the suppression of low quality reads using Trim Galore [34] (requested phred score: 28, minimum read length: 100 bp). Paired-end reads were subsequently merged with pandaseq [35] employing the default configuration to obtain sequences with a typical average length of 262 bp (see Table 1 for variation). Using BLASTN (http://blast.ncbi.nlm.nih.gov), the merged sequences were aligned against a collection of the currently known *dfrB* sequences (Table S2) and against the resfinder [36] database of acquired antibiotic resistance genes available from the Center for Genomic Epidemiology (http://www.genomicepidemiology.org/). Only high quality alignments were retained by requesting a minimum match length of 50 bp and a sequence identity ≥95%. The merged reads were further processed through metaxa2 [37] to analyze the composition of the bacterial community based on 16 S rRNA gene fragments. Reads being tested positive for *dfrB* were also scanned for the signatures of integrons using the IntegronFinder [38] application provided by the Pasteur Institute, France, as a galaxy-based web service (https://galaxy.pasteur.fr). The same reads were also aligned against the ISfinder [39] database of insertion sequences maintained by the Laboratoire de Microbiologie et Génétique Moléculaires, Toulouse, France, using their web service at https://www-is.biotoul.fr/index.php.

#### Assembly and analysis of the flanking regions of *dfrB* genes

Because of the limited read length, it is difficult to identify the genetic context of *dfrB* genes exclusively from original short read data. Consequently, gene context analyses call for metagenome assembly which is challenging, not least due to the exceptionally high demand for computer memory (RAM). Even if the assembly succeeds technically, a substantial extension of the sequences of primary interest is not necessarily guaranteed. This is especially so for low-coverage metagenomes of highly diverse microbial communities [40]. Consequently, we implemented an algorithm that specifically targets the assembly of the flanking regions of *dfrB*-like sequences. Briefly, a collection of the reads giving specific hits for *dfrB* variants was built by an initial run of BLASTN. Those reads served as “seeds” in a subsequent process of iterative sequence extension. In each iteration, the algorithm searches the remaining pool of yet “unused” reads for candidates allowing for a plausible extension of the seed sequence (in iteration 1) or the outcome of earlier iterations according to sequence similarity in overlapping parts (min. 50 bp overlap). The algorithm relies on BLASTN as the workhorse for sequence alignments and, due to the targeted focus on specific seeds, RAM usage remains very low. The source code of the seed-based assembler was made publicly available at https://github.com/dkneis/close2gene together with basic documentation and a minimum working example. Overall, our seed-based approach turned out to be very close to the one implemented in GenSeed [41], a software which we only detected later.

From the assembled contigs, the seeds (i.e., the *dfrB*) were finally removed and the remaining flanking sequences were fed into BLASTX (http://blast.ncbi.nlm.nih.gov) to identify the genetic context of *dfrB* at protein level. The flanking sequences were also scanned for insertion sequences as outlined for the original short reads. Very short assemblies of <250 bp after removal of the *dfrB* seed sequence were excluded from downstream analysis.

### Statistical analysis

#### General methods

Statistical data analysis and visualization was performed in R [42] version 4.2.1. Principal component analysis was performed in base R (prcomp) using power-transformed relative abundances as input (power 0.2). Shifts in locations were assessed by means of the non-parametric Wilcoxon rank sum test (wilcox.test). The significance of differences in odds ratios was analyzed with Fisher’s exact test (fisher.test). Finally, confidence of relative abundance estimates were obtained from an exact binomial test (binom.test). Whenever multiple, logically connected hypotheses were tested simultaneously, the corresponding *p* values were adjusted to control the false discovery rate [43].

#### Correlations between ARGs and 16S rRNA-based species markers

Demonstrating statistical associations between ARG abundances and bacterial community composition is difficult when sample sizes are unequal. The intuitive solution is to employ normalization so as to achieve comparable counts of ARGs and taxonomic markers across samples, that is, the counts are divided by the total number of 16 S rRNA gene copies in the respective sample. While such normalization is very common, it is often overlooked that the obtained relative abundances are unsuitable for correlation analysis [44]. Unfortunately, valid alternatives to correlation like, for example, log ratio analyses, are not applicable either because the number of detected ARG copies is zero in a notable fraction of samples. In view of this challenge, we performed correlation analyses on the original count data (i.e., without normalization) using a downsampling approach to account for unequal sample sizes. Specifically, we identified the 10% quantile of 16 S rRNA gene copies across the considered samples (Q_10_). Samples with fewer than Q_10_ copies were subsequently discarded. For the remaining samples, we studied the correlation between ARG counts and 16 S rRNA gene counts attributed to a certain bacterial group after random-driven truncation to the common length Q_10_. The process was repeated 250 times and the median of the correlation coefficient (Spearman’s rho) was finally evaluated.

## Results

### Unequal distribution of *dfrB* gene variants in river and wastewater

**Fig. 1:**
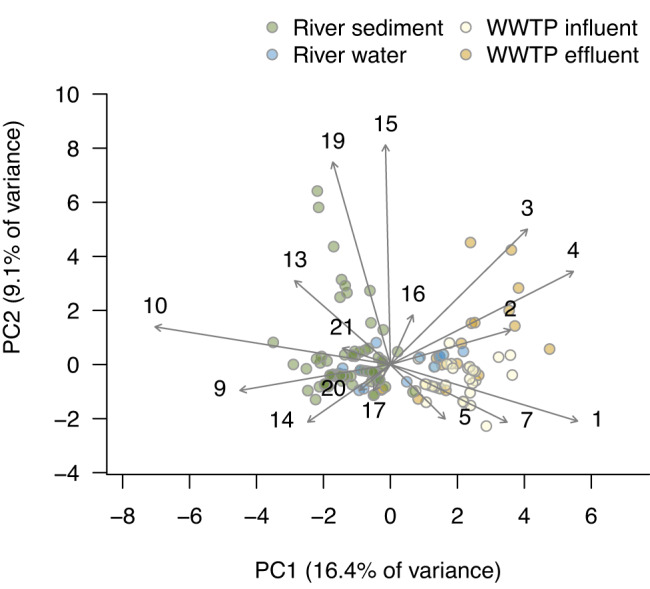
Principal component analysis of the relative abundance of *dfrB* gene variants. Dots indicate individual samples colored by their origin. Integers represent gene variants (e.g., ‘10’ denotes *dfrB10*). See Fig. S1 for the corresponding scree plot.

A principal component analysis suggests the separation of the data into two major subsets based on the abundance of distinct *dfrB* variants (Fig. 1). In particular, the established gene variants with smaller numeric indices (*dfrB1* to *dfrB7*), that were the first to be identified, appear to be associated with wastewater while the recently discovered variants rather cluster with river samples. However, the underlying data structure appears to be more complex since only about 25% of the total variance is resolved by the first two principal components.

**Fig. 2:**
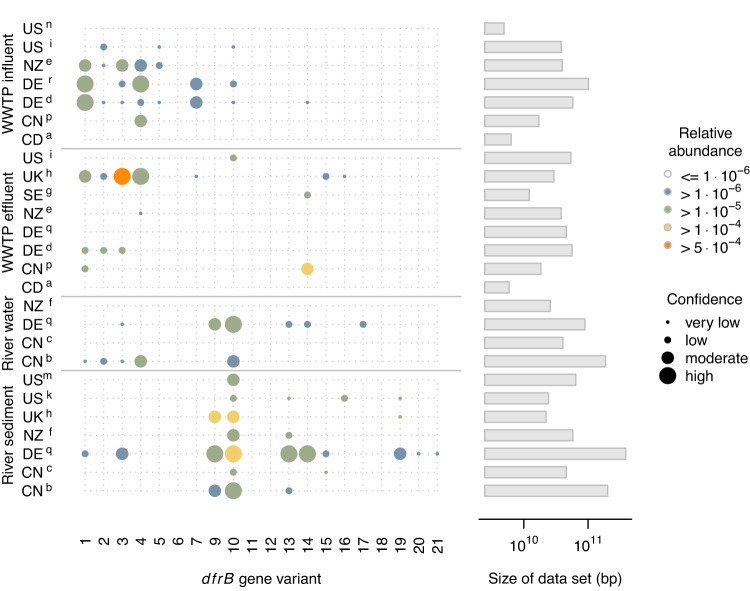
Relative abundance of *dfrB* gene variants (copies/16 S rRNA gene copies) in distinct subsets of samples. Dot size encodes the width of the 95% confidence interval around empirical estimates according to a binomial model (high: <0.5 log units, moderate: <1 log unit, low & very low: >1 and >2 log units, respectively). Capital letters encode countries, superscripts denote studies according to Table 1.

Another graphical representation of the distribution of *dfrB* gene variants across different groups of samples exhibit a number of characteristic patterns (Fig. 2). Whereas some variants were primarily detected in wastewater-borne samples (e.g., *dfrB1, dfrB2*, *dfrB4*, *dfrB7*) others were found primarily or even exclusively in surface water and sediment samples (*dfrB9*, *dfrB10*, *dfrB13*, and *dfrB19*). Four out of the 20 known variants (*dfrB6*, *dfrB11*, *dfrB12*, *dfrB18*) could not be recovered from any of the screened metagenomic DNA reads based on the criterion of 95% sequence identity. For the recovered gene variants, estimated relative abundances frequently exceeded 10^−5^ copies × (16 S rRNA gene copies)^−1^ and four variants (*dfrB3*, *dfrB9*, *dfrB10, dfrB14*) occurred in relative abundances >10^−4^.

The estimated relative abundances of some *dfrB* variants are subject to uncertainty as it is common for rare resistance genes (Fig. 2). In particular, the failure to detect any *dfrB* gene copies in some individual small-sized data sets is most likely a result of undersampling given the limited sensitivity of shotgun metagenomics as compared to quantitative PCR-based approaches. Nevertheless, for all of the groups of samples distinguished in Table 1 and Fig. 2, the number of 16 S rRNA gene copies used as a reference exceeds 1 × 10^6^ such that reported relative abundances are unlikely to suffer from systematic bias (see Fig. S2 for details).

When the data are broadly divided by compartments into just two subsets (wastewater vs. river) many of the contrasts visible in Figs. 1 and 2 pass the threshold of statistical significance (Table 2). This applies to all gene variants which were more frequently found in wastewater communities, that is, *dfrB1* to *dfrB7*. Likewise, the elevated prevalence in environmental samples was confirmed for *dfrB9*, *dfrB10*, and *dfrB13*. To exclude the possibility that the statistics are generally flawed due to global undersampling, we performed significance tests also for split samples. For all but two gene variants (*dfrB5*, *dfrB13*), the reported contrasts in relative abundance between wastewater and river-borne samples were consistently detected in data subsets, each representing 50% of the total analyzed material (Table 2).

**Table 2:** Comparison of the relative abundance of *dfrB* gene variants (copies per 16 S rRNA gene copies) in WWTP effluents and river samples.

Gene variant	Wastewater samples	River samples	Higher in	adj. *p* value	Signif. code	Confirmed by split sampling in
*dfrB1*	3.7e−05	1.8e−06	Wastewater	1.9e−17	***	2/2 subsets
*dfrB2*	3.9e−06	1.2e−06	Wastewater	6.3e−04	***	2/2 subsets
*dfrB3*	6.6e−05	5.9e−06	Wastewater	1.5e−05	***	2/2 subsets
*dfrB4*	1.9e−05	4.7e−06	Wastewater	2.4e−07	***	2/2 subsets
*dfrB5*	1.6e−06	0e+00	Wastewater	0.0064	**	1/2 subsets
*dfrB7*	4.7e−06	0e+00	Wastewater	8.8e−06	***	2/2 subsets
*dfrB9*	0e+00	4.6e−05	River samples	3.5e−06	***	2/2 subsets
*dfrB10*	2.4e−06	2e−04	River samples	1.3e−17	***	2/2 subsets
*dfrB13*	0e+00	2.2e−05	River samples	0.0011	**	1/2 subsets
*dfrB14*	3.5e−06	1.7e−05	River samples	0.26	n.s.	
*dfrB15*	7.8e−07	2.3e−06	River samples	1	n.s.	
*dfrB16*	3.9e−07	1.2e−06	River samples	0.65	n.s.	
*dfrB17*	0e+00	1.2e−06	River samples	0.44	n.s.	
*dfrB19*	0e+00	3.5e−06	River samples	0.14	n.s.	
*dfrB20*	0e+00	5.9e−07	River samples	0.57	n.s.	
*dfrB21*	0e+00	5.9e−07	River samples	0.57	n.s.	

Reported *p* values refer to a Wilcoxon rank sum test with the null hypothesis being that the relative abundances of *dfrB* gene variants are equal in both groups. Each variant was considered a distinct hypothesis and *p* values were adjusted accordingly (***: *p* < 0.001, **: *p* < 0.01, n.s.: not significant). The rightmost column indicates whether the significance of contrasts (adj. *p* value  <  0.05) could be verified on independent data subsets, each representing 50% of the bacterial DNA from wastewater and river samples, respectively.

### Statistical association of *dfrB* with bacterial community composition

Using the subsampling technique outlined in the methods section, we scanned for empirical associations between the frequency of *dfrB* gene variants and the abundance of taxonomically defined bacterial groups (orders). The analysis was carried out separately for river-borne samples and for samples of treated and untreated wastewater. For the river-borne samples, no statistically significant association between the abundance of the predominant gene variants *dfrB9* or *dfrB10* and the abundance of bacterial orders could be identified (Spearman’s rho rarely exceeding 0.15; all *p* values > 0.1). In wastewater samples, however, the abundance of *dfrB3* was found to be most closely associated with five orders of Gram-negative bacteria (*Aeromonadales*, *Chromatiales*, *Pseudomonadales*, *Enterobacterales, Campylobacterales*) all but the last belonging to the class of *γ-Proteobacteria*. The corresponding rank correlation coefficients (Spearman’s rho) reached values in range 0.5–0.8 (*p* < 0.05 each).

### Physical association of *dfr* genes with other genetic markers

#### Analysis of the original short reads

The prospects of full metagenome assembly are limited in the case of short-read environmental samples subject to high microbial diversity [40] while the computational effort is very high. In particular, the probability of detecting ARGs and species markers (such as variable regions of the 16 S rRNA gene) on the same contig are very low and the possibility of assembly errors may limit the confidence in the validity of metagenome-assembled genomes [45]. Consequently, in a first step, we analyzed the immediate genetic neighborhood of the *dfr* fragments on the original (merged) reads. In particular, we scanned all reads containing signatures of *dfr* for the presence of other ARGs which would indicate embedding of the former in resistance gene cassette arrays.

The vast majority of reads where *dfrB* occurred in direct proximity of other ARGs originated from wastewater samples (61 out of 65; Table 3). By contrast, only a minor fraction (4/65) of the reads containing *dfrB* and another ARG was attributed to river-borne samples. Considering the total number of *dfrB* gene copies in both groups (wastewater: 357, river: 550), the likelihood of *dfrB* being found in neighborship with other ARGs was significantly higher in wastewater samples as compared to river samples (OR 0.036 [0.0093–0.097], *p* < 10^−15^, Fisher’s exact test, see Table S3). The majority of such co-occurrences (16 of the 21) was due to the variants *dfrB1*, *dfrB3*, and *dfrB4* being found in direct proximity of *ant* genes coding for aminoglycoside resistance.

**Table 3:** Co-occurrence of *dfrB* and *dfrA* gene fragments with signatures of other antibiotic resistance genes on one and the same read (see Table 1 for average read lengths).

			*dfrB* gene variant					
Target	Linked ARG	Origin	*1*	*3*	*4*	*5*	*7*	*10*
Aminoglyc.	*aac*	WWTP infl.	8/5				2/2	
Aminoglyc.	*aac*	WWTP effl.	1/1					
Aminoglyc.	*ant*	WWTP infl.	8/7		7/2		3/3	2/1
Aminoglyc.	*ant*	WWTP effl.		16/7				
Aminoglyc.	*ant*	River water			3/1			
Ansamycin	*ARR*	WWTP infl.	2/2			?		
Ansamycin	*ARR*	WWTP effl.		?				
Beta-lactam	*blaGES*	WWTP infl.				?		
Beta-lactam	*blaOXA*	WWTP infl.	2/2	?			1/1	
Beta-lactam	*blaOXA*	WWTP effl.			?			
Phenicol	*catB*	WWTP infl.			1/1			
Phenicol	*catB*	WWTP effl.	?		2/1			
Phenicol	*catB*	River water			1/1			

			*dfrA* gene variant												
Target	Linked ARG	Origin	*1*	*5*	*7*	*12*	*13*	*14*	*15*	*16*	*17*	*21*	*22*	*27*	*32*
Aminoglyc.	*aac*	WWTP infl.			2/2	5/4	?				2/2		?		
Aminoglyc.	*aac*	River water				1/1		3/2							?
Aminoglyc.	*aac*	River sedim.				1/1									
Aminoglyc.	*aadA*	WWTP infl.	5/5			2/2		48/31	?		3/3				
Aminoglyc.	*aadA*	WWTP effl.	1/1					108/9							
Aminoglyc.	*aadA*	River water									1/1				
Aminoglyc.	*ant*	WWTP infl.	32/14	10/7	4/4	8/7	?	38/23		5/4	3/3	2/2			
Aminoglyc.	*ant*	WWTP effl.	14/5		1/1	1/1		2/2	4/3	5/4			1/1		
Aminoglyc.	*ant*	River water		2/2	1/1	2/1					1/1		1/1	?	5/5
Aminoglyc.	*aph*	WWTP infl.						4/3							
Ansamycin	*ARR*	WWTP infl.	14/8											2/2	
Ansamycin	*ARR*	WWTP effl.	6/4												
Beta-lactam	*blaGES*	WWTP infl.						?				?			
Beta-lactam	*blaOXA*	WWTP infl.	?					?							
Beta-lactam	*blaOXA*	River water	2/2												
Macrolide	*ere*	WWTP infl.	?	?											
Phenicol	*cmlA*	WWTP infl.						4/2							
Quinolone	*qnrVC*	WWTP infl.						3/2							

Both *dfr* and the linked gene(s) fulfilled the criterion of at least 95% sequence identity. The first integer represents the number of double-positive reads, the integer after the slash indicates the number of distinct samples in which those reads were found. If a particular gene combination was observed only once across the entire data set, numbers were replaced by a question mark to indicate uncertainty associated with those singletons.

For the purpose of comparison, we performed the same analysis for the *dfrA* gene family which is responsible for moderate TMP resistance. The overall results were very similar to those obtained for *dfrB* (Table 3, Table S4). Specifically, *dfrA* was way more likely to be found in neighborship with other ARGs in samples of wastewater as compared to river samples (OR 0.11 [0.070–0.18, *p* < 10^−15^). Likewise, *dfrA* was most commonly associated with genes conferring resistance to aminoglycosides, namely *aad*A and *ant*.

The alignment of *dfrB*-positive short reads against databases of mobile genetic elements yielded a similar outcome as the analysis of resistance gene co-occurrences. In total, six short reads were identified which harbored both a sequence of *dfrB* and a cluster of *attC* recombination sites lacking an integron-integrase. The latter are commonly referred to as CALINs [46] representing degraded or incompletely sequenced integrons [30]. All of these reads originate from samples of treated or untreated wastewater and five out of six cases are attributable to the variants *dfrB2* and *dfrB3* (Table S5).

According to insertion sequence analysis, *dfrB* signatures were almost exclusively found in proximity to a single type of transposable element, Tn*As3* (Table S6), originally discovered in the fish pathogen *Aeromonas salmonicida*. The apparent primary association of *dfrB* with this element is of particular relevance with regard to mobility. According to a recent study [47], an estimated proportion of 70% of Tn*As3* have integrons within their genetic context of 10 ORF and they belong to the most abundant insertion sequences found on plasmids. In our dataset, most of the reads giving a simultaneous hit for both *dfrB* and Tn*As3* originated from wastewater samples (85 out of 90 cases; Table S6) with *dfrB3* again being predominant. We did not observe a single case of Tn*As3* in direct proximity of a *dfrB* with a numeric index >7.

#### Analysis of the assembled flanking regions of *dfrB* genes

The analysis of the assembled flanking regions of *dfrB* genes overall confirms the outcome of the above evaluations based on short reads (Table 4 and S7). For the gene variants *dfrB1* to *dfrB*7, a total of 33 flanking regions with a length >250 bp were recovered (length range 259–1228 bp, excluding the *dfrB* itself). In 27 of 33 cases (80%), significant BLASTX alignments were obtained for either integron integrases (8 cases) or resistance determinants (19 cases), including beta-lactamases, enzymes mediating aminoglycoside resistance, and multi-drug efflux pumps. The clear majority of those flanking regions (31/33) were recovered from wastewater-borne datasets.

**Table 4:** Analysis of the flanking regions of *dfrB1* to *dfrB7* (left panel) and *dfrB* variants with indices >8 (right panel).

Gene	Origin of read	Superfamily	Putative function	IS	Gene	Origin of read	Superfamily	Putative function	IS
*dfrB1*	Wi (DE)	DNA_BRE_C	integron integrase	+	*dfrB9*	Rw (DE)	−	no significant alignment	−
*dfrB1*	Wi (DE)	DNA_BRE_C	integron integrase	+	*dfrB9*	Rs (DE)	−	no significant alignment	−
*dfrB1*	Wi (DE)	DNA_BRE_C	integron integrase	+	*dfrB9*	Rs (DE)	−	no significant alignment	−
*dfrB1*	Wi (NZ)	Ybx1	class D betalactamase	−	*dfrB9*	Rs (DE)	−	no significant alignment	−
*dfrB1*	Wi (DE)	DNA_BRE_C	integron integrase	+	*dfrB9*	Rs (DE)	−	no significant alignment	−
*dfrB1*	Wi (DE)	DNA_BRE_C	integron integrase	+	*dfrB10*	Rs (DE)	CoxL	xanthine dehydrogenase	−
*dfrB1*	Wi (DE)	DNA_BRE_C	integron integrase	+	*dfrB10*	Rs (DE)	−	isoprenylcysteine carboxymethyltransferase	−
*dfrB1*	Wi (DE)	N6_acetyl_AAC6	aminoglycoside acetyltransferase	−	*dfrB10*	Rs (DE)	BrnT_toxin	BrnA antitoxin	−
*dfrB1*	Wi (DE)	ANT_3pp_9_crypt	aminoglycoside nucleotidyltransferase	+	*dfrB10*	Rs (DE)	CoxL	xanthine dehydrogenase	−
*dfrB2*	We (UK)	EamA	multidrug efflux pump	+	*dfrB10*	Rs (UK)	PRK08270	ribonucleoside triphosphate reductase	−
*dfrB2*	Wi (NZ)	−	hypothetical protein	−	*dfrB10*	Rs (NZ)	−	hypothetical protein	−
*dfrB2*	We (UK)	RT_like	reverse transcriptase/maturase	−	*dfrB10*	Rs (DE)	Yads	trimeric intracellular cation channel	−
*dfrB3*	We (UK)	DNA_BRE_C	aminoglycoside resistance	+	*dfrB10*	Rs (DE)	−	hypothetical protein	−
*dfrB3*	Wi (NZ)	YbxI	class D betalactamase	−	*dfrB10*	Rs (DE)	PKc-like	phosphotransferase	−
*dfrB3*	Wi (NZ)	−	hypothetical protein	+	*dfrB10*	Rs (DE)	VapI	addiction module antidote	−
*dfrB3*	We (UK)	EamA	multidrug efflux pump	+	*dfrB10*	Rs (DE)	VapI	addiction module antidote	−
*dfrB3*	Wi (DE)	DUF1010	plasmid-encoded protein of unknown function	−	*dfrB10*	Rs (DE)	−	BrnA antitoxin	−
*dfrB3*	We (UK)	EamA	multidrug efflux pump	+	*dfrB10*	Rw (CN)	−	reverse transcriptase	−
*dfrB3*	We (UK)	EamA	multidrug efflux pump	+	*dfrB10*	Rs (DE)	−	isoprenylcysteine carboxymethyltransferase	−
*dfrB3*	We (UK)	EamA	multidrug efflux pump	+	*dfrB10*	Rs (DE)	PKc-like	phosphotransferase	−
*dfrB3*	We (UK)	EamA	multidrug efflux pump	+	*dfrB10*	Rs (DE)	−	isoprenylcysteine carboxymethyltransferase	−
*dfrB3*	We (UK)	EamA	multidrug efflux pump	+	*dfrB10*	Rs (DE)	−	oxidoreductase	−
*dfrB3*	We (UK)	EamA	multidrug efflux pump	+	*dfrB10*	Rs (DE)	VapI, ParE_toxin	addiction module antidote	−
*dfrB4*	Rw (CN)	DNA_BRE_C	integron integrase	+	*dfrB10*	Rs (DE)	PKc-like	phosphotransferase	−
*dfrB4*	Wi (DE)	EamA	multidrug efflux pump	−	*dfrB10*	Rw (NZ)	Ggt	gamma-glutamyltransferase	−
*dfrB4*	We (UK)	−	no significant alignment	−	*dfrB10*	Rs (NZ)	Imm6, DUF2486	deaminase reductase	−
*dfrB4*	We (UK)	DNA_BRE_C	aminoglycoside resistance	+	*dfrB10*	We (US)	−	hypothetical protein	−
*dfrB4*	Wi (DE)	DNA_BRE_C	integron integrase	+	*dfrB14*	Rs (DE)	HP	histidine phosphatase	−
*dfrB4*	Rw (CN)	LbetaH	acetyltransferase	−	*dfrB15*	Rs (DE)	−	hypothetical protein	−
*dfrB5*	Wi (DE)	−	aminoglycoside acetyltransferase	−	*dfrB15*	Rs (DE)	SSP160	N-acetylmuramoyl-L-alanine amidase	−
*dfrB5*	Wi (US)	transpeptidase	ext. spect. class A betalactamase	+					
*dfrB7*	Wi (DE)	N6_acetyl_AAC6	aminoglycoside acetyltransferase	−					
*dfrB7*	Wi (DE)	N6_acetyl_AAC6, EamA	aminoglycoside acetyltransferase	−					

Listed are protein superfamilies, if conserved domains were detected by BLASTX, as well as associated functions. The column “IS” indicates the detection (+) or absence (−) of signatures of the Tn*As3* transposon by a BLASTN search. See the Table S7 for an extended version of this table holding accession numbers as well as information the length of assemblies and the quality of hits.

*Wi* WWTP influent, *We* WWTP effluent, *Rw* River water, *Rs* River sediment.

By contrast, integron integrases or ARGs were not identified in any of the 30 assembled flanking regions of *dfrB9* (5 cases)*, dfrB10* (22 cases), or variants with a greater numeric index (3 cases). All but one of these assemblies were recovered from river-borne samples. If conserved domains were detectable at all, they were generally indicative of enzymes not directly related to antimicrobial resistance or ARG mobility. For *dfrB10*, our assemblies suggest a number of typical genetic framings as flanks with similar contents were recovered from multiple independent samples (Table S7). Namely xanthine dehydrogenases, phosphotransferases, carboxymethyltransferases, as well as two different components of toxin-antitoxin (TA) systems were observed in multiple contigs originating from distinct samples.

The observed differences in potential mobility of *dfrB* genes as inferred from the detection of integron integrases or ARG in the assembled flanking regions are highly significant. The fact that *dfrB* genes being strongly associated with wastewater metagenomes (*dfrB1* to *dfrB7*) frequently showed indications of mobility (27 of 33 cases) while other *dfrB* variants associated with river metagenomes did not (0 of 30 cases) is very unlikely to be by chance (OR > 23, *p* < 10^−11^; Fisher’s exact test). Strong significance (OR > 5.3, *p* < 10^−6^) remains even if the presence of TA systems in five of the flanking regions of *dfrB10* is generally counted as an indication of mobility, based on the historical notion that TA systems are often plasmid-encoded, which is not necessarily the case [48, 49].

## Discussion

### Prevalence and hosts of the “classical” clinical *dfrB* gene variants

Our data suggest that treated wastewater is a primary source of the “classical” variants of *dfrB* with numeric indices in the range of 1–5 but also 7, which comprise the earliest *dfrB* identified [29, 50, 51]. The relative abundance in wastewater-borne metagenomes was significantly increased for all of those variants as compared to freshwater-borne metagenomes. Some of the variants (*dfrB5*, *dfrB7*) were exclusively recovered from wastewater samples, a fact that should not be overinterpreted, however, considering the limited sensitivity of metagenomics as compared to PCR in the detection of low abundance genes [52]. Overall, the analyzed dataset supports the notion that the classical variants of *dfrB* are primarily harbored by human gut-associated bacteria.

In correlation analysis, members of the phylum *γ-Proteobacteria*, including, for example, *Aeromonadales* and *Pseudomonadales*, emerged as the most likely candidate host of one of the gene variants, *dfrB3*. As usual, however, correlation provides indications rather than rigorous evidence. In particular, the identification of definite candidate hosts is naturally limited by collinearities, arising from the natural structure of microbial communities. The sharing of common niches or the formation of metabolic networks, for example, inevitably lead to substantial correlations between distinct bacterial groups which may not be disentangled in host identification analysis. Nevertheless, the proposed primary association of the classical *dfrB* variants with *γ-Proteobacteria* is very plausible, as it perfectly agrees with the outcome of whole genome-based studies [28].

In the analysis of the original short read data, most of the classical *dfrB* variants, especially *dfrB1*, *dfrB3*, and *dfrB4*, were also found in direct proximity to other ARGs (Table 3). Considering the type of the linked ARGs, our findings corroborate the outcome of earlier WGS-based studies according to which the earliest reported *dfrB* genes are mostly (but not exclusively) associated with genes mediating resistance to aminoglycosides [28, 53]. In this respect, these *dfrB* variants are similar to genes of the *dfrA* family. The rare linkage of *dfrB* with genes mediating resistance to phenicol, betalactam (*bla*OXA), and rifampicin (*arr*) found in the analyzed metagenomes is also consistent with former WGS-based findings [28]. The direct neighborship of *dfrB1* to *dfrB7* to other ARGs was further confirmed by the evaluation of the assembled flanking regions. In addition to enzymes providing resistance against aminoglycosides and betalactams, multi-drug efflux pumps were frequently detected in proximity to the dihydrofolate reductases (Table 4).

The frequent co-occurrence of the classical *dfrB* variants with other ARGs suggests that a notable percentage of the gene copies is embedded in resistance cassette arrays as has been demonstrated by WGS analysis [28, 29, 53]. It can further be hypothesized that the respective *dfrB* genes are potentially part of mobile genetic elements like integrons and transposons facilitating their proliferation, including plasmid-based horizontal transfer. The alignment of *dfrB*-positive short reads against integron and insertion sequences databases strongly supports this hypothesis of mobility. Specifically, all of the 90 cases where a *dfrB* gene was found in direct proximity to an insertion sequence, most typically Tn*As3*, were attributable to the gene variants *dfrB1* to *dfrB7* (Table S6). Similarly, integron signatures were almost exclusively found in neighborship with these wastewater associated *dfrB* variants (Tables 4 and S5).

### Occurrence of recently discovered *dfrB* genes

The recently described gene variants, especially *dfrB9* to *dfrB21*, were discovered in a heterogeneous set of sequences comprising both bacterial isolate genomes [28] and metagenomes of environmental origin [30]. However, owing to the limited number of *dfrB*-positive sequences studied so far, it was previously impossible to infer quantitative information on the occurrence and distribution of these gene variants. Based on consistently processed metagenomic datasets, our study provides quantitative information on the abundance of *dfrB9* to *dfrB21* in complex environmental communities, which is a key to uncovering possible anthropogenic impacts.

According to our analysis, the variants *dfrB9*, *dfrB10*, *dfrB13*, and *dfrB14* were shown to be significantly more prevalent in freshwater environments than in wastewater. Hence, very likely, these gene variants are primarily hosted by environmental bacteria which do not play a predominant role in municipal wastewater systems. This finding is consistent with the observation that many of the genes mediating clinically relevant antibiotic resistance have their origin in environmental bacteria [54] which has now been confirmed for a broad variety of genes and hosts [55–57]. However, the original role of many ancestral antibiotic resistance genes is still unknown and different factors may have shaped their evolution in environmental bacteria [58, 59]. From the anthropocentric perspective, the resistance trait is often at the center of interest and the presence of ARGs in antibiotic producers or their interspecific competitors is indeed expected from an ecological point of view. However, in the particular case of TMP, no natural analogues are known to date. Hence, the original selective advantage associated with environmental *dfrB* genes like, for instance, *dfrB9* and *dfrB10* is unrelated to self-resistance or microbial chemical warfare. The ecological advantage leading to the maintenance of these genes is yet to be identified. For example, the encoded DfrB could exhibit a favorable metabolic costs profile in the host compared to alternatives dihydrofolate reductases like the ubiquitous FolA. However, the *dfrB* genes could also be subject to selection by yet unknown factors unrelated to dihydrofolate reduction.

So far, information on actual bacterial hosts of *dfrB* genes is largely confined to the variants *dfrB1* to *dfrB5* and mostly pertains to genomes of human-related isolates [28]. The variant *dfrB10* was originally discovered in a typical soil bacterium, *Pseudomonas putida*, involved in the infection of a patient in China [60]. Beyond that, we are not aware of successful attempts to disclose the bacterial hosts of, for example, *dfrB9* to *dfrB21* in environmental communities which inspired us to screen for statistical correlations between *dfrB* abundance and community composition. However, even the most prominent environmental gene variant in our data set, *dfrB10*, was not significantly associated with any bacterial order such that no host candidates could be identified. This could reflect the presence of *dfrB10* in a variety of species due to horizontal transfer of, for example, plasmid-borne instances or the conservation over a long evolutionary history. However, a lack of close statistical associations would also be observed if *dfrB10* was actually linked to a primary host which is just too rare to be adequately represented in communities inferred from amplification-free shotgun metagenomics.

According to our analyses, the genetic context of the recently described *dfrB* genes appears to deviate substantially from the one identified for *dfrB1* to *dfrB7*. For example, none of the sequences hosting *dfrB9* to *dfrB21* was associated to insertion sequences, neither in the analysis of short reads, nor in the evaluation of assembled flanks. Similarly, according to our data set, genotypic multiple-resistance involving *dfrB9* to *dfrB21* appears to be a rare exception as it was observed in a single metagenome only where, as opposed to the general trend, *dfrB10* was recovered from a wastewater sample. These findings agree with genetic context analyses of environmental sequences in which *dfrB11* to *dfrB21* were originally identified [30]. We conclude from the available direct and indirect evidence that, as opposed to *dfrB1* - *dfrB7*, the recently discovered *dfrB* variants, are less likely associated with mobile elements which are recognized as major facilitators of the spread of clinically relevant antimicrobial resistance [61]. Nevertheless, this must not be misinterpreted in the sense that the newly identified *dfrB* variants are generally immobile and thus pose a lower risk compared to other TMP resistance genes. In particular, *dfrB10* was originally discovered on a plasmid [60] and our data set demonstrates rare cases of combined TMP-aminoglycoside resistance involving *dfrB10*. Also *dfrB9* was earlier detected within a plasmid-based integron in a clinical isolate classified as *Enterobacteriaceae* and other novel *dfrB* variants were found in the context of CALINs previously [30] (*dfrB12*, *dfrB19*) or in the present study (*dfrB15*). Hence, clear indications exist for actual and potential mobility of the recently discovered *dfrB* variants. Consequently, we have to expect that, under ambient conditions selecting for TMP resistance, these variants can undergo a fast spread by horizontal gene transfer in addition to vertical proliferation.

**Fig. 3:**
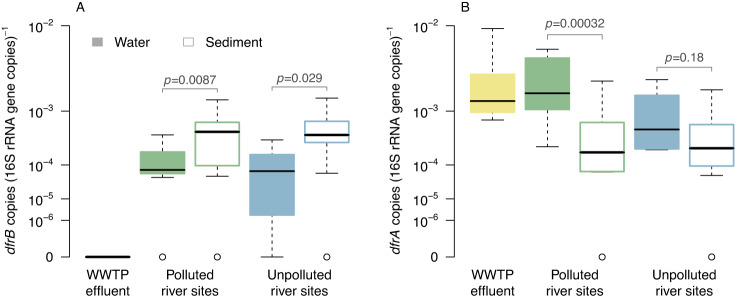
Relative abundance of *dfrB* and *dfrA* genes aggregated at family level in samples from the Lockwitzbach River Basin, Germany (study label “q” in Table 1). Panel **A** refers to the *dfrB* gene and panel **B** to *dfrA*. The *p* values refer to a shift in location between water and sediment-borne samples (Wilcoxon rank sum test). Note the custom *y*-axis resulting from power transformation with an exponent of 0.2.

Besides emissions from sewage disposal, many surface waters receive additional anthropogenic inputs of bacteria linked to livestock farming and runoff from organically fertilized soils [62]. We cannot exclude that the occurrence of the environment-associated variants like *dfrB9* or *dfrB10* reflects such agricultural inputs. However, our data do not comprise any evidence for such a hypothesis. First, if the detection of the mentioned *dfrB* variants was reflecting emissions from livestock farming under TMP-selective conditions, we would expect them to commonly appear in neighborship with other resistance genes or mobile elements just like their wastewater-borne counterparts *dfrB1* to *dfrB7*. This was not the case. Moreover, copies of *dfrB10*, *dfrB13*, *dfrB16*, and *dfrB19* were also recovered from sequences which are very unlikely to be impacted by intensive farming. This applies in particular to the river sediment metagenomes from the US-based Great Smoky Mountains national park and unpolluted sites considered in one the datasets from Germany. Finally, if the occurrences of *dfrB* genes in river-borne samples was determined by contamination from external sources, we would expect to find the genes preferably in the water phase mediating the transport. However, in the most comprehensive dataset (label “q” in Table 1) which comprises water and sediment-borne samples, we rather see the opposite trend (Fig. 3A). The relative abundance of *dfrB* genes is significantly higher in sediment-borne DNA compared to the water-borne DNA at both polluted and unpolluted sampling sites. This is in contrast to *dfrA* where the higher relative abundance in the water phase as compared to sediment reflects a contamination of sampling sites exposed to WWTP effluents (green boxes Fig. 3B).

### Implications for AMR research and management

Our findings suggest that the classical *dfrB* gene variants like *dfrB1* or *dfrB4* are established in human-associated bacterial communities as signaled by their primary association with wastewater metagenomes. Hence, together with members of the *dfrA* family, these *dfrB* variants appear to actively contribute to contemporary, clinically relevant bacterial resistance against TMP. By contrast, gene variants like *dfrB9*, *dfrB10*, or *dfrB13* were rarely detected in an anthropogenic context, yet they appear to be present in many freshwater systems around the world. The respective aquatic bacterial communities thus represent a reservoir of additional TMP resistance that is potentially mobilizable and could further compromise the treatment of bacterial infections in the future.

**Fig. 4:**
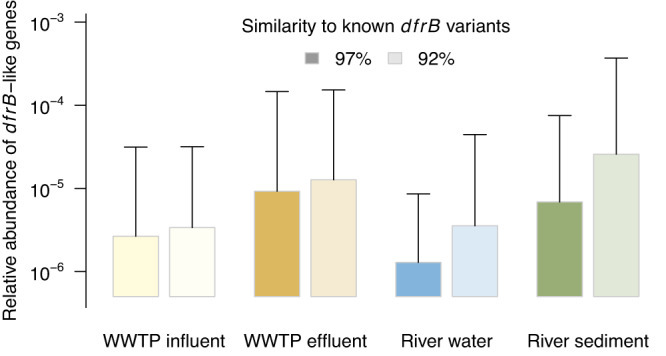
Effect of the chosen similarity threshold on the relative abundance of *dfrB*-like sequences identified in datasets of different origin. Bars represent averages, whiskers illustrate maximum values.

The full spectrum of *dfrB* genes present in environmental bacterial communities is yet to be disclosed. A list of 20 new candidate *dfrB* genes was released only recently [30] and the metagenomes screened in this study suggest a considerable potential for new gene discoveries as well. This can be demonstrated by comparing the abundance of the presently known *dfrB* to the abundance of *dfrB*-like sequences satisfying a relaxed criterion of sequence identity (Fig. 4). Especially river-borne community DNA contains a notable amount of additional *dfrB*-like sequences, some of which may turn out to be novel TMP resistance genes in future analyses.

From a research-centered perspective, we propose to work toward a more comprehensive understanding of the distribution of *dfrB* across ecosystems or geographic regions. Ideally, the respective samples would be acquired, processed, and sequenced according to common protocols so as to exclude methodology-borne biases in comparisons between data subsets. Standardization would need to cover, for example, DNA extraction protocols, library preparation, read length, or sequencing depth and should also consider sample barcoding schemes that reduce the chance of misclassification (e.g., through unique dual indexing). Metagenomic sequencing should further be complemented by a more sensitive quantification of *dfrB* via quantitative or digital PCR to better discriminate actually low gene abundances from undersampling effects. Likewise, we suggest fostering the identification of the respective bacterial hosts and the genetic framing to improve our understanding of the evolutionary trajectories of the different *dfrB* variants as well as possible pathways of past and future proliferation. The assembly of long-read metagenomic sequences [63, 64] might be a viable approach toward improved host characterization but it certainly remains challenging in view of the low relative abundance of *dfrB* and the substantial diversity of natural bacterial communities. Plasmid-capturing [65] and subsequent sequencing would be a possible means to specifically target *dfrB* genes of high lateral mobility. Our study demonstrates that the prevalence of antimicrobial resistance genes must be examined at the level of particular gene variants. A more aggregated analysis at the *dfrB* gene family level, for instance, would not have resolved the existing contrasts in gene distributions.

Overall, our study highlights the possibility of omnipresent environmental genes being promoted into a threat for human society. Thus, the concentrations of antibiotics to which environmental communities are exposed must be held below selective thresholds [66] not only to control the spread of established ARGs but also to prevent the rise of yet unknown genes hidden in environmental communities. Our study demonstrates that a promotion of such unknown resistance genes can be triggered even by synthetic drugs, like TMP, where natural analogs had not been an original selection factor.

## Supplementary information

Supplement (main file)

Supplement table S1

Supplement table S7

## Data Availability

All sequence data analyzed are publicly available in the sequence read archive (https://www.ncbi.nlm.nih.gov/sra). See Table S1 for the respective accession numbers.

## References

[1] Gleckman R, Blagg N, Joubert DW (1981). Trimethoprim: mechanisms of action, antimicrobial activity, bacterial resistance, pharmacokinetics, adverse reactions, and therapeutic indications. Pharmacother J Hum Pharmacol Drug Ther.

[2] Amyes SG (1989). The success of plasmid-encoded resistance genes in clinical bacteria. An examination of plasmid-mediated ampicillin and trimethoprim resistance genes and their resistance mechanisms. J Med Microbiol.

[3] Crellin E, Mansfield KE, Leyrat C, Nitsch D, Douglas IJ, Root A (2018). Trimethoprim use for urinary tract infection and risk of adverse outcomes in older patients: cohort study. BMJ.

[4] Quinlivan EP, McPartlin J, Weir DG, Scott J (2000). Mechanism of the antimicrobial drug trimethoprim revisited. FASEB J.

[5] World Health Organization. Model list of essential medicines - 22nd List. WHO reference number: WHO/MHP/HPS/EML/2021.02. (2021).

[6] Somorin YM, Weir N-JM, Pattison SH, Crockard MA, Hughes CM, Tunney MM (2022). Antimicrobial resistance in urinary pathogens and culture-independent detection of trimethoprim resistance in urine from patients with urinary tract infection. BMC Microbiol.

[7] Vellinga A, Tansey S, Hanahoe B, Bennett K, Murphy AW, Cormican M (2012). Trimethoprim and ciprofloxacin resistance and prescribing in urinary tract infection associated with *Escherichia coli*: a multilevel model. J Antimicrob Chemother..

[8] Pouwels KB, Freeman R, Muller-Pebody B, Rooney G, Henderson KL, Robotham JV (2018). Association between use of different antibiotics and trimethoprim resistance: going beyond the obvious crude association. J Antimicrob Chemother.

[9] Barth PT, Datta N, Hedges RW, Grinter NJ (1976). Transposition of a deoxyribonucleic acid sequence encoding trimethoprim and streptomycin resistances from R483 to other replicons. J Bacteriol..

[10] Kahlmeter G, Poulsen HO (2012). Antimicrobial susceptibility of *Escherichia coli* from community-acquired urinary tract infections in Europe: the ECO·SENS study revisited. Int J Antimicrob Agents.

[11] Fasugba O, Mitchell BG, Mnatzaganian G, Das A, Collignon P, Gardner A (2016). Five-year antimicrobial resistance patterns of urinary *Escherichia coli* at an Australian tertiary hospital: Time series analyses of prevalence data. PloS ONE.

[12] Sundqvist M, Geli P, Andersson DI, Sjölund-Karlsson M, Runehagen A, Cars H (2010). Little evidence for reversibility of trimethoprim resistance after a drastic reduction in trimethoprim use. J Antimicrob Chemother..

[13] Queener SF, Cody V, Pace J, Torkelson P, Gangjee A (2013). Trimethoprim resistance of dihydrofolate reductase variants from clinical isolates of *Pneumocystis jirovecii*. Antimicrob Agents Chemother..

[14] Zinner SH & Mayer KH. Sulfonamides and trimethoprim; trimethoprim-sulfamethoxazole. in Mandell, Douglas, and Bennett’s Principles and Practice of Infectious Diseases, 9th ed. In: Bennett JE, Dolin R & Blaser MJ, editors. 416–425.e3. ISBN 9780323482554 (Elsevier, 2020).

[15] Damier-Piolle L, Magnet S, Brémont S, Lambert T, Courvalin P (2008). AdeIJK, a resistance-nodulation-cell division pump effluxing multiple antibiotics in *Acinetobacter baumannii*. Antimicrob Agents Chemother..

[16] Huovinen P (2001). Resistance to trimethoprim-sulfamethoxazole. Clin Infect Dis Off Publ Infect Dis Soc Am..

[17] Sánchez-Osuna M, Cortés P, Llagostera M, Barbé J, Erill I (2020). Exploration into the origins and mobilization of di-hydrofolate reductase genes and the emergence of clinical resistance to trimethoprim. Microb Genom.

[18] Sköld O, Widh A (1974). A new dihydrofolate reductase with low trimethoprim sensitivity induced by an R factor mediating high resistance to trimethoprim. J Biol Chem..

[19] Amyes SGB, Smith JT (1974). R-factor trimethoprim resistance mechanism: An insusceptible target site. Biochem Biophys Res Commun..

[20] Pattishall KH, Acar J, Burchall JJ, Goldstein FW, Harvey RJ (1977). Two distinct types of trimethoprim-resistant dihydrofolate reductase specified by R-plasmids of different compatibility groups. J Biol Chem..

[21] Mayer KH, Fling ME, Hopkins JD, O’Brien TF (1985). Trimethoprim resistance in multiple genera of *Enterobacteriaceae* at a U.S. hospital: spread of the type II dihydrofolate reductase gene by a single plasmid. J Infect Dis..

[22] Alcock BP, Raphenya AR, Lau TTY, Tsang KK, Bouchard M, Edalatmand A (2020). CARD 2020: antibiotic resistome surveillance with the comprehensive antibiotic resistance database. Nucleic Acids Res..

[23] Thungapathra M, Amita, Sinha KK, Chaudhuri SR, Garg P, Ramamurthy T (2002). Occurrence of antibiotic resistance gene cassettes aac(6′)-Ib, dfrA5, dfrA12, and ereA2 in class I integrons in non-O1, non-O139 *Vibrio cholerae* strains in India. Antimicrob Agents Chemother..

[24] Sáenz Y, Briñas L, Domínguez E, Ruiz J, Zarazaga M, Vila J (2004). Mechanisms of resistance in multiple-antibiotic-resistant *Escherichia coli* strains of human, animal, and food origins. Antimicrob Agents Chemother..

[25] Welch TJ, Fricke WF, McDermott PF, White DG, Rosso M-L, Rasko DA (2007). Multiple antimicrobial resistance in plague: an emerging public health risk. PloS ONE.

[26] Howell EE (2005). Searching sequence space: two different approaches to dihydrofolate reductase catalysis. Chembiochem Eur J Chem Biol.

[27] Lemay-St-Denis C, Alejaldre L, Jemouai Z, Lafontaine K, St-Aubin M, Hitache K (2023). A conserved SH3-like fold in diverse putative proteins tetramerizes into an oxidoreductase providing an antimicrobial resistance phenotype. Philos Trans R Soc B Biol Sci..

[28] Lemay-St-Denis C, Diwan S-S, Pelletier JN (2021). The bacterial genomic context of highly trimethoprim-resistant DfrB dihydrofolate reductases highlights an emerging threat to public health. Antibiotics.

[29] Toulouse JL, Edens TJ, Alejaldre L, Manges AR, Pelletier JN (2017). Integron-associated DfrB4, a previously uncharacterized member of the trimethoprim-resistant dihydrofolate reductase B Family, is a clinically identified emergent source of antibiotic resistance. Antimicrob Agents Chemother..

[30] Cellier-Goetghebeur S, Lafontaine K, Lemay-St-Denis C, Tsamo P, Bonneau-Burke A, Copp JN (2022). Discovery of highly trimethoprim-resistant DfrB dihydrofolate reductases in diverse environmental settings suggests an evolutionary advantage unrelated to antibiotic resistance. Antibiotics.

[31] Jadeja NB, Worrich A (2022). From gut to mud: dissemination of antimicrobial resistance between animal and agricultural niches. Environ Microbiol..

[32] Karkman A, Pärnänen K, Larsson DGJ (2019). Fecal pollution can explain antibiotic resistance gene abundances in anthropogenically impacted environments. Nat Commun..

[33] Dolejska M, Literak I (2019). Wildlife is overlooked in the epidemiology of medically important antibiotic-resistant bacteria. Antimicrob Agents Chemother..

[34] Krueger F. A wrapper tool around Cutadapt and FastQC to consistently apply quality and adapter trimming to FastQ files. (2015). https://github.com/FelixKrueger/TrimGalore.

[35] Masella AP, Bartram AK, Truszkowski JM, Brown DG, Neufeld JD (2012). PANDAseq: paired-end assembler for illumina sequences. BMC Bioinformatics.

[36] Zankari E, Hasman H, Cosentino S, Vestergaard M, Rasmussen S, Lund O (2012). Identification of acquired antimicrobial resistance genes. J Antimicrob Chemother..

[37] Bengtsson-Palme J, Hartmann M, Eriksson KM, Pal C, Thorell K, Larsson DGJ (2015). metaxa2: Improved identification and taxonomic classification of small and large subunit rRNA in metagenomic data. Mol Ecol Resour..

[38] Néron B, Littner E, Haudiquet M, Perrin A, Cury J, Rocha EPC (2022). IntegronFinder 2.0: Identification and analysis of integrons across bacteria, with a focus on antibiotic resistance in Klebsiella. Microorganisms.

[39] Siguier P, Perochon J, Lestrade L, Mahillon J, Chandler M (2006). ISfinder: the reference centre for bacterial insertion sequences. Nucleic Acids Res.

[40] Kneis D, Berendonk TU, Forslund SK, Heß S (2022). Antibiotic resistance genes in river biofilms: A metagenomic approach toward the identification of sources and candidate hosts. Environ Sci Technol..

[41] Sobreira TJP, Gruber A (2008). Sequence-specific reconstruction from fragmentary databases using seed sequences: implementation and validation on SAGE, proteome and generic sequencing data. Bioinformatics.

[42] R Core Team. R: A language and environment for statistical computing. (R Foundation for Statistical Computing, Vienna, Austria, 2022).

[43] Benjamini Y, Hochberg Y (1995). Controlling the false discovery rate: a practical and powerful approach to multiple testing. J R Stat Soc Ser B.

[44] Lovell D, Pawlowsky-Glahn V, Egozcue JJ, Marguerat S, Bähler J (2015). Proportionality: A valid alternative to correlation for relative data. PLOS Comput Biol..

[45] Shaiber A, Eren AM (2019). Composite metagenome-assembled genomes reduce the quality of public genome repositories. mBio.

[46] Cury J, Jové T, Touchon M, Néron B, Rocha EP (2016). Identification and analysis of integrons and cassette arrays in bacterial genomes. Nucleic Acids Res.

[47] Razavi M, Kristiansson E, Flach C-F, Larsson DGJ (2020). The association between insertion sequences and antibiotic resistance genes. mSphere.

[48] Heaton BE, Herrou J, Blackwell AE, Wysocki VH, Crosson S (2012). Molecular structure and function of the novel BrnT/BrnA toxin-antitoxin system of *Brucella abortus*. J Biol Chem..

[49] Díaz-Orejas R, Espinosa M, Yeo CC (2017). The Importance of the expendable: toxin–antitoxin genes in plasmids and chromosomes. Front Microbiol..

[50] Kadlec K, Kehrenberg C, Schwarz S (2005). Molecular basis of resistance to trimethoprim, chloramphenicol and sulphonamides in *Bordetella bronchiseptica*. J Antimicrob Chemother.

[51] Szczepanowski R, Linke B, Krahn I, Gartemann K-H, Gützkow T, Eichler W (2009). Detection of 140 clinically relevant antibiotic-resistance genes in the plasmid metagenome of wastewater treatment plant bacteria showing reduced susceptibility to selected antibiotics. Microbiology.

[52] Heß S, Kneis D, Österlund T, Li B, Kristiansson E, Berendonk (2019). Thomas U. Sewage from airplanes exhibits high abundance and diversity of antibiotic resistance genes. Environ Sci Technol..

[53] Pérez-Viso B, Hernández-García M, Ponce-Alonso M, Morosini MI, Ruiz-Garbajosa P, del Campo R (2021). Characterization of carbapenemase-producing *Serratia marcescens* and whole-genome sequencing for plasmid typing in a hospital in Madrid, Spain (2016–18). J Antimicrob Chemother.

[54] D’Costa VM, King CE, Kalan L, Morar M, Sung WWL, Schwarz C (2011). Antibiotic resistance is ancient. Nature.

[55] Ebmeyer S, Kristiansson E, Larsson DGJ (2019). PER extended-spectrum β-lactamases originate from *Pararheinheimera* spp. Int J Antimicrob Agents.

[56] Ebmeyer S, Kristiansson E, Larsson DGJ (2019). The mobile FOX AmpC beta-lactamases originated in *Aeromonas allosaccharophila*. Int J Antimicrob Agents.

[57] Kieffer N, Ebmeyer S, Larsson DGJ (2022). Evidence for *Pseudoxanthomonas mexicana* as the recent origin of the *blaAIM-1* carbapenemase gene. Int J Antimicrob Agents.

[58] Sengupta S, Chattopadhyay MK, Grossart H-P (2013). The multifaceted roles of antibiotics and antibiotic resistance in nature. Front Microbiol..

[59] Allen HK, Donato J, Wang HH, Cloud-Hansen KA, Davies J, Handelsman J (2010). Call of the wild: antibiotic resistance genes in natural environments. Nat Rev. Microbiol..

[60] Sun F, Zhou D, Wang Q, Feng J, Feng W, Luo W (2016). Genetic characterization of a novel *blaDIM-2*-carrying megaplasmid p12969-DIM from clinical *Pseudomonas putida*. J Antimicrob Chemother..

[61] Partridge SR, Tsafnat G, Coiera E, Iredell JR (2009). Gene cassettes and cassette arrays in mobile resistance integrons. FEMS Microbiol Rev..

[62] Amos GC, Gozzard E, Carter CE, Mead A, Bowes MJ, Hawkey PM (2015). Validated predictive modelling of the environmental resistome. ISME J.

[63] Bickhart DM, Watson M, Koren S, Panke-Buisse K, Cersosimo LM, Press MO (2019). Assignment of virus and antimicrobial resistance genes to microbial hosts in a complex microbial community by combined long-read assembly and proximity ligation. Genome Biol.

[64] Moss EL, Maghini DG, Bhatt AS (2020). Complete, closed bacterial genomes from microbiomes using nanopore sequencing. Nat Biotechnol..

[65] Smalla K, Heuer H, Götz A, Niemeyer D, Krögerrecklenfort E, Tietze E (2000). Exogenous isolation of antibiotic resistance plasmids from piggery manure slurries reveals a high prevalence and diversity of IncQ-like plasmids. Appl Environ Microbiol..

[66] Kraupner N, Ebmeyer S, Hutinel M, Fick J, Flach C-F, Larsson DGJ (2020). Selective concentrations for trimethoprim resistance in aquatic environments. Environ Int..

