# Supplementary material for: Trimethoprim resistance in surface and wastewater is mediated by contrasting variants of the dfrB gene
Source: ISME J. 2023 Jun 27;17(9):1455–66. doi: 10.1038/s41396-023-01460-7 (PMC10432401; doi:10.1038/s41396-023-01460-7)
Supplement: Supplementary file 1 — Supplement (main file) [file 41396_2023_1460_MOESM1_ESM.pdf]

Trimethoprim resistance in surface and wastewater is mediated by contrasting variants of the *dfrB* gene

(Kneis et al., 2023, ISME J)

Supplementary material

Accession numbers and metadata of the analyzed metagenomes

Table S1: Complete accession and metainformation on the analyzed metagenomes

This table is provided in tab-delimited text format as a separate supplementary file.

Query sequences of the studied *dfrB* variants

Table S2: Nucleotide sequences of the currently known variants *dfrB* variants which passed verification of phenotypic TMP resistance. The sequences of *dfrB10* and *dfrB11* were first reported by Lemay-St-Denis et al., 2021, DOI: 10.3390/antibiotics10040433. The sequences of *dfrB12* and beyond were presented by Cellier-Goetghebeur et al., 2022, DOI: 10.3390/antibiotics11121768 as protein sequences only.

dfrB1	ATGGAACGAAGTAGCAATGAAGTCAGTAATCCAGTTGCTGGCAATTTTGTATTCCCATCGAACGCCACGT<br>TTGGTATGGGAGATCGCGTGCGCAAGAAATCCGGCGCCGCCTGGCAAGGTCAGATTGTCGGGTGGTACTG<br>CACAAATTTGACCCCCGAAGGCTACGCCGTCGAGTCTGAGGCTCACCCAGGCTCAGTACAGATTTATCCT<br>GTTGCGGCGCTTGAACGCATCAACTGA
dfrB2	ATGGGTCAAAGTAGCGATGAAGCCAACGCTCCCGTTGCAGGGCAGTTTGCGCTTCCCCTGAGTGCCACCT<br>TTGGCTTAGGGGATCGCGTACGCAAGAAATCTGGTGCCGCTTGGCAGGGTCAAGTCGTCGGTTGGTATTG<br>CACAAAACTCACTCCTGAAGGCTATGCGGTCGAGTCCGAATCCCACCCAGGCTCAGTGCAAATTTATCCT<br>GTGGCTGCACTTGAACGTGTGGCCTAA
dfrB3	ATGGACCAACACAACAATGGAGTCAGTACTCTAGTTGCTGGCCAGTTTGCGCTCCCATCGCACGCCACGT<br>TTGGCCTGGGAGATCGCGTGCGCAAGAAATCTGGCGCCGCTTGGCAGGGTCAAGTTGTCGGGTGGTACTG<br>CACAAAACTGACCCCTGAAGGCTATGCCGTCGAGTCCGAGTCTCACCCCGGTTCAGTACAGATTTATCCT<br>GTGGCTGCGCTTGAACGCGTGGCCTGA
dfrB4	ATGAATGAAGGAAAAAATGAGGTCAGTACTTCAGCTGCTGGCCGGTTCGCATTCCCATCAAACGCCACGT<br>TTGCCTTGGGGGATCGCGTACGCAAGAAGTCTGGCGCTGCTTGGCAGGGGCGCATTGTCGGGTGGTACTG<br>CACAACACTTACCCCTGAAGGCTACGCCGTCGAGTCCGAATCTCACCCAGGCTCAGTCCAGATTTATCCC<br>ATGACTGCGCTTGAACGGGTGGCCTGA
dfrB5	ATGGACCAAGGCAGAAGTGAAGTCAGTAATCCAGTTGCTGGCCAGTTTGCGTTCCCTTCAAACGCCGCGT<br>TCGGAATGGGAGATCGCGTGCGCAAGAAATCTGGCGCCGCTTGGCAAGGCCAGATTGTCGGGTGGTACTG<br>CACAAAATTGACCCCTGAAGGGTACGCTGTCGAGTCTGAGGCTCACCCTGGCTCGGTACAGATTTATCCT<br>GTTGCGGCACTGGAACGCATCAACTGA
dfrB6	ATGGACCAAGGTAGCAATGAAGTCATTAATCCAGTCGCTGGCCAGTTTGCGTCCCCATCGAACGCCACGT<br>TTGGTATGGGAGATCGCGTGCGCAAGAAATCTGGCGCCGCCTGGCAAGGTCAGATTGTCGGGTGGTACAG<br>CACAAAGTTGACCCCTGAAGGCTACGCTGTCGAGTCTGAGGCTCACCCTGGCTCGGTGCAGATTTATCCT<br>GTTGCCGCGCTTGAACGCGTCAACTGA

dfrB7	ATGGACCAAGGTAGCAATGAAGTCGGTAATCCAGTTGCGGGCCAGTTTTCGTTCCCATCGAACGCCGCGT<br>TTAGTATGGGAGATCGCGTGCGCAAGAAATCGGGCGCCGCTTGGCAAGGTCAGATTGTCGGGTGGTACTG<br>CACAAAGTTGACCCCTGAAGGCTACGCTGTCGAGTCTGAGGCTCACCCTGGCTCGGTACAGATTTATCCT<br>GTTGCGGCGCTTGAACGCATCAACGGAGTTCAAGGTTGA
dfrB9	ATGAATCAAAGTAGCAATTGCATCAGCACTCCAGTTGTTGGACAGTTTGCGCTGCCATTTCAACCCACGT<br>TCGGCCTGGGAGATCGCGTACGCAAGAAGTCTGGCGCCGCTTGGCAAGGTAAAGTTGTCGGCTGGTACTG<br>CACAAAATTAACCCCTGAAGGCTACGCGGTCGAGTCCGAAGCTCATCCAGGCTCAGTGCAGATTTATCCT<br>GTGGCTGCGCTTGAACGCGTGGCCTAA
dfrB10	ATGGATCAAAGTAGCAATGAAGTCAGCACTCCAGTTGCTGGCCAGTTTGCGCTCCCATTGCGCGCCACGT<br>TTGGCCTGGGAGATCGCGTACGCAAGAAATCTGGCGCCGCTTGGCAAGGTCAAGTTGTCGGCTGGTACTG<br>CACAAAACTGACCCCTGAAGGCTATGCAGTCGAGTCCGAGTCTCACCCAGGCTCAGTACAGATTTATCCT<br>GTGGCTGCGCTTGAACGCGTGGCCTAA
dfrB11	ATGGATCAAAGTAGTAAAGAGGTTGGCACTCCCGTTGTTGGCCAGTTTGCACTCCCGTCGCACGCCACGT<br>TTGGCCTTGGAGACCGCGTTCGCAAGAAATCGGGCGCCGCTTGGCAGGGTCAAGTTGTGGGCTGGTATTG<br>CACAAAGCTGACCCCTGAAGGCTATGCCGTCGAGTCCGAGTCTCACCCAGGCTCGGTACAAATTTATCCA<br>GTGAATGCGCTTGAACGCGTGGCCTGA
dfrB12	ATGAAACAGAGTAGCGATGGCCTAGGTGCTCCGATTGATCACCAGTTTGCGCTGCCGCTAACCGCCGTGT<br>TCAGGCTGGGCGATCGCGTTCGCAAGAAGTCTGGCTCCGCGTGGCAGGGCCGAATCGTCGGCTGGTACAG<br>CACGAAGTTGACGCCTGAGGGCTATGCCGTTGAGTCCGACGCCCACCCGGGCTCGGTACATATTTACCCA<br>GTCGCGGCGCTTGAGCGCGTGGCCTAA
dfrB13	ATGGGACAAATCAGCAAAGTCGTTGGTTCTCAAAATGAGCGCCAACTCAAGCAGCCAGAACCGACGACGT<br>TTGGACTAGGAGATCGAGTACGAAAAAAGTCCGGGGCCGCGTGGCAAGGCTGCGTCGTTGGCTGGTACAG<br>CACAACTCTGACTCCAGAGGGTTATGCCGTCGAGTCCGAGGCTCATCCAGGCTCAGTACAAATCTACCCG<br>GTCGCGGCACTTGAACGCGTGGACTAA
dfrB14	ATGGATCAAAGTGGCAATCAAGTCAGAACCCCAGTCGGTGACCAGCTTGCGCTCCCATCGCGCGCCACGT<br>TTGGCCTGGGAGATCGAGTGCGCAAGAAATCTGGCGCCGCTTGGCAGGGGCACGTTGTCGGCTGGTACTG<br>CACGAAACTGACCCCTGAGGGCTATGCCGTGGAGTCCGAGTTTCACCCAGGCTCAGTACAGATCTATCCT<br>GTGACTGCGCTTGAACGCGTGCCCTAA
dfrB15	ATGGCTCAAAGCAGCAATGACAGCAGTTCTTCAGTTGCTAGCGCGCTTGGGCTCCCTTCGAACGCCACGT<br>TTCGTCTGGGAGATCGGGTACGCAAGAAGTCTGGCGCCGCCTGGCAAGGACATATTGTCGGCTGGTACTG<br>CACGAGCCTGACCCCGGAAGGTTATGCCGTCGAGTCAGAGTCTCATGCGGGCTCAGTACAGATCTATCCT<br>GTTGCTGCACTTGAGCGCGTGGTCTAG
dfrB16	ATGGAACAGAGTAACGGTGCCGCCAAAGCTTCACACGATCCTCAGTTTCAATTGCCAACGCCAGCGACGT<br>TTGGGTTGGGAGAACGCGTTCGCAAAAAGTCGGGCGCCGCGTGGCAAGGCAATGTCGTCGGGTGGTACAG<br>CACGAAACTGACGCCAGAGGGATACGCCGTCGAATCCGAAGCACATCCAGGCTCGGTGCAAATCTACCCG<br>GCCGCGGCGCTTGAGCGCGTGGCTTAA
dfrB17	ATGGAAGAGACAGGCAATGATGTCGGCAATTTAGTTGGGCGCCAGTTTGCGCTGCCCTTGAGCGCCGTGT<br>TTGGGCTAGGCGATCTCGTTCGCAAAAGGTCTGGTGCCGCGTGGCAGGGTCGAGTAGTCGGCTGGTACAG<br>CACGCAACTGACTCCTGAAGGGTATGCCGTTGAGTCAGAAGCTCACCCAGGGTCGGTGCAGATCTACCCG<br>GTCGCGGCGCTGGAACGCGTGGCCTAA
dfrB18	ATGAATACCGATAGCAAGCACGCGAGCGCTGCGCTCGCCGCGCAGTTTGCGCTGCCAGCCGGCGCTACGT<br>TCGCCTTGGGCGATCGCGTCCGCAAGAAGTCGGGCGCCGCATGGCAAGGGCTCGTCGTGGGCTGGTACAG<br>CACGAAGCTCACGGCCGAAGGCTACGCGGTCGAATCCGAGGCGCACCCGGGTTCCGTGCAGATCTATCCC<br>GTCGCTGCGCTCGAACGCGTCGCCTGA

dfrB19	ATGGAACAGAGTAGCGTTGGCTTTGGAGCTTCAAACGGTAATCAGATTCAACAGCAAATGACAGCCACGG<br>TTGGGTTGGGAGAACGCGTTCGCAAAAAGTCGGGCGCCGCGTGGCAAGGCCACATCGTCGGCTGGTACAG<br>CACGAAGCTGACGCCAGAGGGCTATGCCGTCGAATCTGAAGCACATCCAGGCTCGGTACAGATCTACCCG<br>GTCGCAGCGCTTGAGCGCGTGGCCTAA
dfrB20	ATGAATCAGAATAGCAATGACGTCATTGCTCCCGTTGATGGCCGGCTTGCCCTGCCATTGCGCGCTGCGT<br>TTGGGCTGGGAGATCGCGTACGCAAGAAGTCTGGCGCCGCTTGGCAAGGTCGCGTTGTCGGCTGGTACAG<br>CACGGAACTGACCCCTGAAGGCTATGCCGTTGAATCCGAAGCTCACCCGGGCTCAGTACAGATTTACCCG<br>GTTGGTGCGCTTGAGCCCGTGGCCTAA
dfrB21	ATGGATCGAAGTAGCAATGACGTCAGTACTCCAGTTACCGGTCAGTTTGCGCTGCCGTCGCGCGCCACGT<br>TCAGCTTGGGAGATCGTGTGCGCAAGAAGTCTGGCGCCGCGTGGCAGGGTCGAGTGGTCGGCTGGTACTG<br>CACGAAACTGACCCCCGAAGGCTATGCGGTCGAGTCCGAGTCTCACCCAGGCTCGGTTCAAATCTATCCC<br>GTGGCTGCGCTTGAGCGCGTGGCCTGA

Tabular outputs of auxiliary data analyses

Table S3: Contingency illustrating the likelihood of *dfrB* genes occurring on the same (short) read as other ARGs dependent on the origin of the respective samples (see Table 3 for higher resolution with regard to gene variants).

	Number of reads with a significant hit for		
Origin of sample	*dfrB* and another ARG	*dfrB* only	Total
River water or sediment	4	546	550
Treated or untreated wastewater	61	296	357
Total	65	842	

Table S4: Contingency illustrating the likelihood of *dfrA* genes occurring on the same (short) read as other ARGs dependent on the origin of the respective samples (see Table 3 for higher resolution with regard to gene variants).

	Number of reads with a significant hit for		
Origin of sample	*dfrA* and another ARG	*dfrA* only	Total
River water or sediment	22	1067	1089
Treated or untreated wastewater	349	1936	2285
Total	371	3003	

Table S5: Results of the alignment of *dfrB*-positive short reads against the "integronFinder" integron database being accessible through the galaxy web service of the Pasteur Institute, France, at https://galaxy.pasteur.fr (https://doi.org/10.7490/f1000research.1114334.1). The analysis was conducted in March 2023. The reported e-value is the minimum for the detected *attC* sites within a sequence. Only records with e-values < 1e-3 were considered as potential hits.

Gene variant	Origin of sample	Element detected	e-value
dfrB2	WWTP effluent (DE)	Cluster of *attC* sites lacking integrase nearby (CALIN)	4e-5
dfrB2	WWTP influent (US)	Cluster of *attC* sites lacking integrase nearby (CALIN)	7e-4
dfrB2	WWTP influent (US)	Cluster of *attC* sites lacking integrase nearby (CALIN)	3e-12
dfrB3	WWTP effluent (UK)	Cluster of *attC* sites lacking integrase nearby (CALIN)	2e-7
dfrB3	WWTP effluent (UK)	Cluster of *attC* sites lacking integrase nearby (CALIN)	2e-7
dfrB15	WWTP effluent (UK)	Cluster of *attC* sites lacking integrase nearby (CALIN)	7e-10

Table S6: Results of the alignment of *dfrB*-positive short reads against the "ISfinder" database of insertion sequence being accessible through the web service of the Laboratoire de Microbiologie et Génétique Moléculaires based in Toulouse, France, at https://www-is.biotoul.fr/index.php (analysis conducted in March 2023). Integers represent the number of co-occurrences of particular transposable elements and *dfrB* genes on short reads; empty cells indicate no detection. For *dfrB* genes not included in the table, no co-occurrences were observed.

Transposable element	Origin of samples	*dfrB1*	*dfrB2*	*dfrB3*	*dfrB4*	*dfrB5*	*dfrB7*
Tn*As3*	WWTP influent	11			4	1	2
Tn*As3*	WWTP effluent		1	64	1		
Tn*As3*	River water				4		
Tn*As3*	River sediment	1					
IS*15*	WWTP influent	1					

Table S7: Extended version of Table 4 providing details on the assembled flanking regions of *dfrB*.

This table is provided in spreadsheet format as a separate supplementary file.

Supplementary figures

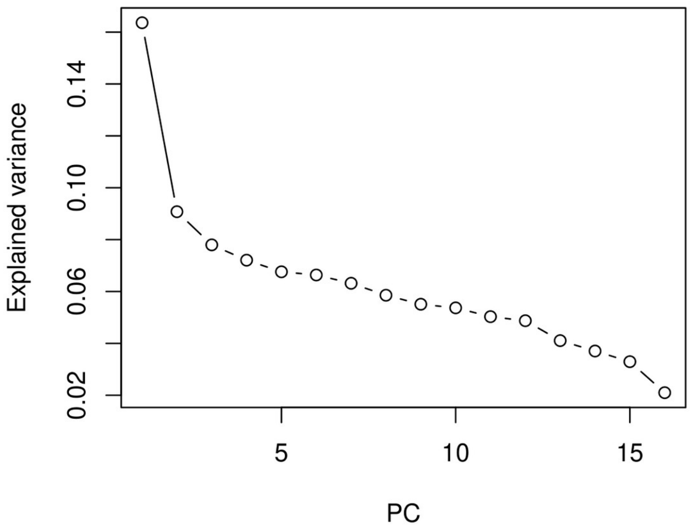

Figure S1: Scree plot corresponding to the principal component analysis from Fig. 1.

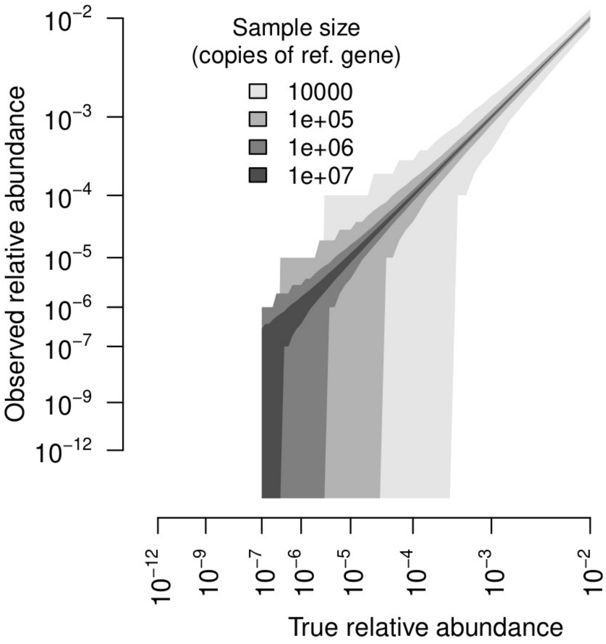

Figure S2: Expected uncertainty in the estimates of relative gene abundance. Shaded areas represent 95% confidence intervals given the indicated sample sizes in terms of the number of 16S rRNA gene copies.
